# Exploring the Impact of Professional Acting on Empathy Development in Medical Students

**DOI:** 10.12688/mep.21228.1

**Published:** 2025-09-19

**Authors:** Nino Shiukashvili, Gvantsa Vardosanidze, Mariam Rochikashvili, Nino Tevzadze, Archil Undilashvili, Mary Jo Lechowicz, Eka Ekaladze

**Affiliations:** 1 Ken Walker International University, Tbilisi, 0141, Georgia; 2 Emory University, Atlanta, Georgia, 30322, USA

**Keywords:** Empathy, Empathy Training, Medical Education, Undergraduate Medical Education, Communication skills, Actor-Led Intervention, Simulated-Based Education

## Abstract

**Background:**

Empathy is essential to patient-centered care and is linked to improved satisfaction, adherence, and clinical outcomes. Yet, empathy often declines during medical training, and traditional teaching methods may fall short in cultivating observable empathic behaviors. This study evaluated a structured, actor-led training program designed to enhance third-year medical students’ empathetic communication.

**Methods:**

Eighteen third-year students participated in a four-week, performance-based workshop incorporating role-play, character immersion, and feedback from professional actors and faculty. Empathetic communication was assessed pre- and post-intervention using a 28-item observational checklist across five domains. Paired-sample t-tests were used to evaluate changes.

**Results:**

Twelve students completed the full training. Statistically significant improvements were observed across all domains (p < 0.001), with the largest gains in verbal expressiveness, non-verbal behavior, and integration of self. All participants showed individual progress, with no significant gender-based differences. The intervention was well-received and deemed feasible for curricular integration.

**Conclusions:**

Actor-led, experiential training significantly enhances medical students’ ability to express empathy through verbal and non-verbal behaviors. These findings support incorporating performance-based methods into undergraduate medical education to foster more emotionally attuned clinicians.

## Introduction

In an era where artificial intelligence is transforming clinical practice, the human capacity for empathy remains essential to patient-centered care, safeguarding relational and emotional dimensions that technology cannot replicate. Empathy is not merely an internal emotional state of the physician, but a patient-perceived experience of being genuinely acknowledged, heard, and understood ( Archer & Meyer, 2021; Hojat *et al.*, 2011). Patient-perceived empathy has been consistently associated with improved satisfaction, adherence to treatment, and clinical outcomes ( Derksen *et al.*, 2013; Mercer & Reynolds, 2002), including in emotionally complex fields such as oncology, chronic illness, and primary care ( Stepien & Baernstein, 2006).

Empathy is most impactful when conveyed through observable behaviors. Several researches demonstrate that specific verbal and non-verbal cues—such as tone of voice, eye contact, posture, and facial expressions—strongly influence patients’ perceptions ( Riess *et al.*, 2012; Silverman, 2009). This externalization of empathy enables clinicians to translate internal understanding into relational connection through the “integration of self”—the ability to bring authenticity into clinical interactions—which has been shown to deepen trust and strengthen therapeutic relationships ( Kelm *et al.*, 2014). Therefore, fostering empathy in medical students requires moving beyond theoretical understanding toward the intentional development of observable and assessable communication behaviors ( Hemmerdinger *et al.*, 2007).

Despite widespread recognition of its value, empathy tends to decline during medical training—particularly during the transition to clinical years—due to increased workload, emotional fatigue, and decreased emphasis on relational learning ( Chen *et al.*, 2007; Neumann *et al.*, 2011). Conventional teaching methods such as reflective writing and patient narratives, while beneficial, may insufficiently address the behavioral and expressive dimensions of empathy ( Batt-Rawden *et al.*, 2013).

To address these limitations, emerging pedagogical approaches now integrate the arts—especially drama and performance—as tools for empathy cultivation. Arts-based methods promote perspective-taking and emotional regulation through experiential immersion ( Shapiro & Rucker, 2003). Acting techniques that focus on character embodiment and emotional attunement have shown promise in medical training for enhancing interpersonal sensitivity and communication ( Wear & Zarconi, 2008). A recent model by Haider *et al.* (2024) further emphasizes that patients perceive empathy largely through clinicians’ verbal and non-verbal behaviors, regardless of their internal affective state, supporting the need for behaviorally oriented training.

Accordingly, we implemented a performance-based training intervention, led by professional actors, to enhance the empathetic communication skills of third-year medical students. This study sought to determine (1) whether the intervention led to measurable improvements in empathic communication and (2) which specific behavioral domains exhibited the most significant gains.

## Methods

### Participants

Sixty third-year medical students from Ken Walker International University were invited to participate in a pilot empathy training program. The university’s six-year MD curriculum includes a pre-medical humanities-focused first year, followed by two years of biomedical sciences, and a clinical phase during the final three years. Third-year students were selected for this study as they had completed foundational training in patient communication, including history-taking and standardized patient encounters.

Eighteen students enrolled on a voluntary basis and were divided into three groups of six. These students participated in a four-week extracurricular workshop. The first session included orientation and baseline empathy assessment, while the following three weeks comprised two sessions per week: an actor-led training workshop and a faculty supervised practice session.

Every student provided written informed consent prior to participation. Ethical approval for the study was obtained from the Ken Walker International University Institutional Review Board, and all procedures were conducted in accordance with the Declaration of Helsinki.

### Intervention design

Each week introduced a new emotionally complex clinical scenario, with students alternating roles between physician and patient. The actor-led sessions focused on verbal and non-verbal aspects of communication—tone, posture, facial expression, and gestures. After guided instruction, students engaged in peer role-play and received immediate, personalized feedback from the professional actor.

In the second session of each week, the same scenario was revisited under faculty observation. Students again rotated roles, receiving structured feedback from the faculty physician, peers, and the actor. This multi-source feedback process reinforced skill development across sessions.

### Assessment

Students’ communication skills were assessed before and after the program using the Empathetic Communication Assessment Form, a checklist adapted from Dow *et al.* (2007), which evaluates verbal and non-verbal behaviors across five communication domains. Each item was scored on a 10-point Likert scale (1 = very poor to 10 = excellent).

The final role-play sessions were independently assessed by both the professional actor and faculty physician using the same checklist as the baseline. This allowed for a direct comparison of pre- and post-intervention performance.

## Results

Eighteen students enrolled in the intervention, but six were excluded due to incomplete attendance, resulting in a final sample of twelve third-year medical students (58.3% female; mean age = 21.25, SD = 0.75). None had prior acting experience. All twelve participants provided complete data for every outcome; no values were missing.

**Figure 1.**
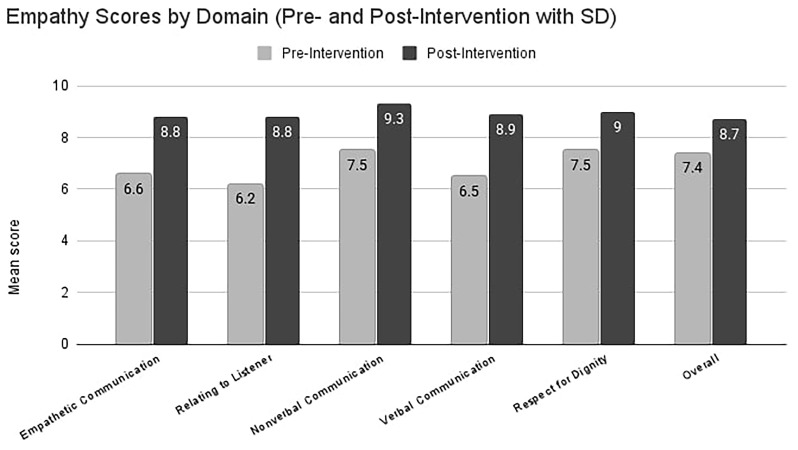
Mean change in empathy-domain scores before and after training. Light-grey bars show the pre-intervention mean (± 1 SD) for each domain in 12 medical students; hatched dark-grey bars show the post-intervention mean (± 1 SD) after the four-week actor-led workshop. Numeric labels above each bar give the exact group mean. Post-intervention scores were significantly higher than pre-intervention in every domain (paired two-tailed t-tests, all p < 0.001).

Paired-sample t-tests showed statistically significant improvements across all domains of empathetic communication (p < 0.001). The largest gains were observed in *Relating to the Listener* (mean difference = +2.6), *Verbal Communication* (+2.4), and *Empathetic Communication* (+2.2). Improvements in *Non-verbal Communication*, *Respect for Dignity*, and the *Overall Score* were also significant ( Table 1; Figure 1).

**Table 1.** Domain-level of Pre- and Post-Test Mean Scores.

Domain	Pre-test Mean (SD)	Post-test Mean (SD)	Mean Diff (Post-Pre)	t-value	df	p-value
Empathetic communication	6.6 (0.85)	8.8 (0.92)	+2.2	-8.6	11	<0.001
Relating to Listener	6.2 (1.03)	8.8 (1.22)	+2.6	-7.7	11	<0.001
Non-verbal Communication	7.5 (0.98)	9.3 (0.87)	+1.8	-5.4	11	<0.001
Verbal Communication	6.5 (0.96)	8.9 (0.92)	+2.4	-9.7	11	<0.001
Respect for Dignity	7.5 (0.83)	9.0 (0.70)	+1.5	-5.3	11	<0.001
Overall	7.4 (0.88)	8.7 (0.94)	+1.3	-5.1	11	<0.001

Domain-level mean (± SD) empathy-communication scores for the 12 participating students before and after the four-week actor-led workshop; positive mean differences indicate improvement (paired two-tailed t-tests, p < 0.05 considered significant; SD = standard deviation, df = degrees of freedom).

All 28 checklist items showed significant score increases, including vocal expressiveness, emotional integration, and positioning relative to the patient. The most substantial change was in “integration of self,” which rose from 5.1 to 8.7 (t = -9.03, p < 0.001) ( Table 2).

**Table 2.** Average Pre- and Post-intervention Scores on Domain-Specific Questions.

Questions	Pre- intervention Mean (SD)	Post- intervention Mean (SD)	t-test	p-value
**Empathetic communication**				
1. Social sensitivity: displays recognition of differences in ethnicity, gender, cognition, etc	7.5 (0.98)	9.7 (0.39)	-8.13	<0.001
2. Patience: allows time for full interpersonal interaction	6.8 (1.52)	8.8 (1.25)	-4.14	0.002
3. Tactfulness: displays the ability to recognize and compensate for patient's feelings	6.1 (1.07)	8.5 (1.15)	-4.58	<0.001
4. Enthusiasm: displays energy and interest in the topic and/or interpersonal relationship	6.0 (1.81)	8.3 (1.19)	-5.19	<0.001
5. Listening while speaking: displays an ability to recognize and adapt to patient's cognition	6.9 (1.47)	8.8 (1.29)	-3.97	0.002
6. Integration of self: displays ability to integrate elements of own personality into interactions (being self, natural, individual)	5.1 (1.62)	8.7 (1.03)	-9.03	<0.001
7. Synthesis: integrates an array of emotional, attitudinal, intellectual, and behavioral qualities	5.9 (1.86)	8.7 (1.24)	-4.90	<0.001
**Relating to Listener**				
8. Integrates multiple modes of communication (visual, aural, kinesthetic)	5.7 (1.47)	8.7 (1.17)	-5.70	<0.001
9. Chooses appropriate language/uses medical language as appropriate	7.1 (0.71)	9.1 (1.17)	-6.61	<0.001
10. Incorporates patient's questions and responses into discourse	5.8 (1.73)	8.8 (1.37)	-6.10	<0.001
11. Checks for non-verbal signs of comprehension	6.1 (1.32)	8.5 (1.53)	-5.69	<0.001
12. Is aware of patient's breath as reflective of emotional state or physical comfort level	6.1 (1.65)	8.6 (1.66)	-6.82	<0.001
**Non-Verbal Communication**				
13. Allows for eye contact	8.0 (1.23)	9.3 (1.22)	-2.99	0.012
14. Presents an open body posture	7.8 (0.89)	9.4 (0.70)	-5.27	<0.001
15. Appropriately positions self in relation to patient (proximity, level, bearing, etc)	6.9 (1.21)	9.3 (0.96)	-6.10	<0.001
**Verbal Communication**				
16. Articulates words clearly	7.0 (1.17)	9.2 (0.93)	-8.18	<0.001
17. Chooses effective vocal placement (timbre, tone, color, etc)	6.5 (1.57)	8.7 (1.14)	-3.83	0.003
18. Projects appropriately/chooses appropriate volume, vocal size	6.1 (1.62)	8.9 (0.90)	-7.16	<0.001
19. Selects appropriate pace	6.4 (1.22)	8.8 (1.08)	-6.33	<0.001
20. Provides appropriate wait time for responses after asking questions	6.6 (1.18)	8.8 (1.30)	-3.74	0.003
21. Varies delivery approach (e.g pitch, rate, emphasis, etc)	6.0 (1.30)	8.9 (1.10)	-6.29	<0.001
**Respect for Dignity**				
22. Establishes an environment respectful of patient confidentiality	8.2 (0.95)	9.5 (0.57)	-4.84	<0.001
23. Clearly describes illness, condition, and/or treatment	7.9 (0.83)	9.3 (0.96)	-3.47	0.005
24. Solicits active patient participation and involvement in care as appropriate	6.8 (1.47)	9.0 (1.14)	-6.44	<0.001
25. Asks permission before touching or intruding on privacy or personal space	6.7 (1.75)	8.2 (1.56)	-2.60	0.025
**Overall**				
26. Presence	7.4 (1.15)	9.2 (0.88)	-4.82	<0.001
27. Attentiveness	7.8 (0.84)	9.2 (0.84)	-6.27	<0.001
28. Physical expressiveness	6.9 (1.48)	8.4 (1.38)	-2.77	0.018

Item-level performance within each empathy-communication domain, showing pre- and post-intervention means (± SD) for individual checklist questions and corresponding paired t-tests (n = 12; bold p-values denote statistical significance at p < 0.05; SD = standard deviation).

Each participant showed statistically significant individual gains (range: +0.8 to +3.1). No gender-based differences were found ( Table 3).

**Table 3.** Comparison of Individual Students’ Pre and Post-Intervention Mean Scores.

Student N	Mean Diff (Post-Pre)	t-value	df	p-value
1	+3.1	-11.5	28	<0.001
2	+2.8	-9.4	28	<0.001
3	+1.1	-7.0	28	<0.001
4	+1.9	-5.2	28	<0.001
5	+2.2	-7.6	28	<0.001
6	+2.9	-11.8	28	<0.001
7	+2.2	-10.0	28	<0.001
8	+2.3	-12.8	28	<0.001
9	+2.6	-11.6	28	<0.001
10	+1.0	-7.2	28	<0.001
11	+0.8	-2.8	28	0.008
12	+1.7	-6.0	28	<0.001

Change in overall empathy-communication score for each student on the 28-item checklist, expressed as mean difference (Post − Pre), associated t-value, and significance level (df = 28 for all comparisons; negative t-values reflect higher post-test scores).

## Discussion

This study demonstrated that a structured, actor-led empathy training program significantly improved third-year medical students’ communication across all assessed domains, including verbal and non-verbal behaviors, emotional responsiveness, and integration of self. These findings reinforce the value of performance-based learning in fostering not just the conceptual understanding of empathy, but its practical, observable expression in clinical settings.

Gains in non-verbal behaviors such as eye contact, body posture, and vocal modulation indicate enhanced physical awareness and intentionality in patient encounters. The marked increase in "integration of self" suggests that experiential training can help students bring authenticity to clinical roles, potentially reducing emotional detachment often associated with early clinical exposure.

These results align with previous literature emphasizing the teachability of empathy through structured, reflective, and feedback-rich experiences ( Batt-Rawden *et al.*, 2013; Riess *et al.*, 2012). By incorporating character immersion and dual role-play, the intervention provided students with an emotionally safe space to explore difficult scenarios and develop nuanced interpersonal responses. Unlike traditional approaches focused primarily on cognitive empathy or written reflection, this training offered embodied practice of empathy as a relational act.

The consistency of improvement across all participants suggests the intervention is broadly applicable regardless of baseline communication ability or gender. Although female students showed slightly higher average gains, the difference was not statistically significant, highlighting the inclusivity of the approach.

Despite these promising findings, several limitations warrant consideration. The small sample size and absence of a control group limit generalizability. Additionally, the voluntary nature of participation introduces potential self-selection bias, as students predisposed to valuing communication may have been more likely to enroll. Finally, while immediate improvements were documented, the long-term retention and real-world clinical application of these skills remain to be assessed.

Given the observed benefits and high feasibility, the program will be integrated into the formal third-year curriculum, coinciding with the transition from pre-clinical to clinical training.

## Conclusion

This study shows that actor-led empathy training significantly enhances medical students’ ability to convey empathy through both verbal and non-verbal behaviors. Performance-based methods provide a valuable, scalable approach to cultivating emotionally attuned and communicatively skilled physicians. Future studies should examine whether these improvements persist longitudinally and translate into enhanced patient care during real-world clinical practice.

## Ethics approval

This study received ethics approval from Biomedical Research Ethics Committee at Ken Walker International University (Approval Number: #1-2024/001). All procedures complied with institutional guidelines and the principles of the Declaration of Helsinki.

## Data Availability

All underlying data, extended materials, and the STROBE checklist are deposited in the Open Science Framework at https://doi.org/10.17605/OSF.IO/U3RBX. All files are available under the Creative Commons Attribution 4.0 International (CC BY 4.0) license. Available through the OSF link above: Empathy domain data: Participant-level pre- and post-scores for five empathy domains (Empathetic Communication, Relating to Listener, Non-verbal, Verbal, Respect to Dignity), with calculated differences and percentage changes. Empathy individual pre-post data: Item-level Empathetic Communication scores for participants (IDs 1–12) before and after the intervention, plus summary statistics. Empathy survey: Image of the 28-item empathy checklist administered to students. Empathy training protocol: Week-by-week outline of the acting-based empathy training programme. STROBE checklist: Completed STROBE reporting checklist. The study follows STROBE recommendations for observational research; the completed checklist is available through OSF.
