# Exploring the Impact of Professional Acting on Empathy Development in Medical Students

**DOI:** 10.12688/mep.21228.3

**Published:** 2026-04-06

**Authors:** Nino Shiukashvili, Gvantsa Vardosanidze, Mariam Rochikashvili, Nino Tevzadze, Archil Undilashvili, Mary Jo Lechowicz, Eka Ekaladze

**Affiliations:** 1 Ken Walker International University, Tbilisi, 0141, Georgia; 2 Emory University, Atlanta, Georgia, 30322, USA

**Keywords:** Empathy, Empathy Training, Medical Education, Undergraduate Medical Education, Communication skills, Actor-Led Intervention, Simulated-Based Education

## Abstract

**Background:**

Empathy is central to patient-centred care and professional identity, yet medical students’ empathy often declines as they transition into clinical training. Theatre-based, arts-and-humanities interventions have been proposed to support empathic communication, but are usually evaluated with self-report rather than performance-based measures.

**Methods:**

We conducted a single-institution, single-group pre–post pilot evaluation of an extracurricular, four-week theatre-based empathy programme for third-year medical students. All 60 students were eligible; 18 volunteered on a first-come, first-served basis, and 12 who attended all eight sessions and completed both assessments formed the analytic sample. The programme, co-facilitated by a professional actor and clinician, was grounded in Kolb’s experiential learning cycle and applied theatre principles. Teaching methods included repeated doctor–patient role-plays in breaking-bad-news scenarios, alternating doctor/patient perspectives, and structured feedback on communication and emotional presence. Observable empathic communication behaviours in simulated consultations were measured before and after the programme using the Empathetic Communication Assessment Form (five domains, 10-point scale), rated by the faculty member and actor. Pre–post differences were analysed with paired-samples t-tests and within-subject effect sizes (Cohen’s d).

**Results:**

Students (n = 12) showed significant improvements across all domains. Mean increases ranged from +1.3 to +2.6 points on the 10-point scale, with large effect sizes (Cohen’s d ≈ 1.5–2.8). The largest gains were in Empathetic Communication (+2.2), Relating to the Listener (+2.6) and Verbal Communication (+2.4). All students improved in their overall checklist score (range +0.8 to +3.1).

**Conclusions:**

This small, single-group pilot suggests that a brief, theatre-based, pre-clerkship programme co-facilitated by a professional actor and clinician may enhance observable empathic communication behaviours in simulated breaking-bad-news encounters. Although limited by the small, self-selected sample and absence of a control group, the findings support further, larger-scale and longitudinal evaluation of theatre-based empathy teaching within arts-and-humanities-informed medical curricula.

## Introduction

Empathy is widely regarded as a core component of patient-centred care and professional identity in medicine. Empathic engagement has been associated with improved patient satisfaction, adherence and clinical outcomes, as well as enhanced clinician well-being and reduced burnout. ^1, 2^ At the same time, longitudinal and cross-sectional studies indicate that medical students’ empathy often declines or stagnates as they progress from preclinical to clinical training, with particularly marked changes around the start of clerkships. ^3^ This pattern has raised concerns that traditional curricula may not adequately support students in sustaining empathic attitudes and behaviours in the face of increasing workload, emotional demands and exposure to clinical realities.

In health care, empathy is commonly conceptualised as a multidimensional construct that includes cognitive understanding of the patient’s perspective, affective attunement to the patient’s emotions and the behavioural expression of this understanding in communication and decision-making. ^4^ This multidimensional view aligns with competencies expected of graduating doctors: accreditation and competency frameworks such as those of the Association of American Medical Colleges (AAMC) and the UK General Medical Council (GMC) emphasise patient-centred care, effective communication and professionalism as core outcomes of undergraduate training, and explicitly or implicitly link these domains to empathic engagement.

Over the past two decades, arts and humanities have increasingly been integrated into health professions education as one of the responses to these expectations. Engagement with literature, visual arts, history, philosophy and performance has been reported to foster perspective-taking, critical reflection, tolerance of ambiguity and empathic understanding, while also supporting learner resilience and well-being. The AAMC’s Fundamental Role of the Arts and Humanities in Medical Education (FRAHME) initiative positions arts and humanities as essential, not optional, for educating physicians who can integrate scientific knowledge with emotional intelligence, social awareness and ethical sensitivity, and calls for more rigorous evaluation of arts- and humanities-based curricula across the continuum of medical education. ^5^ Recent work further emphasises the importance of including artist–practitioners alongside medical educators through co-designed curricula and collaborative research and publication with those who deliver the artistic components. ^6^ Nevertheless, many programmes still offer relatively brief or fragmented teaching about empathy in the early years, with less structured support at later stages when students assume greater clinical responsibility. ^7^

Within this broader movement, theatre-based pedagogies have attracted particular interest because they bring together embodiment, role-play, narrative and immediate feedback in a single modality. Drawing on principles of experiential learning, applied theatre and reflective practice, collaborations between medical schools and theatre professionals have produced interventions ranging from improvisation workshops to scripted and forum theatre. These interventions aim to enhance awareness of non-verbal cues, emotional presence and empathic responses in challenging encounters, and are generally associated with high learner satisfaction, perceived gains in empathy and communication skills, and improvements in self-reported empathy scores. ^8, 9^ However, most theatre-based and other empathy-focused interventions in medical education are evaluated primarily through self-report instruments and qualitative or narrative feedback rather than through structured observation of empathic communication behaviours. ^10– 12^ While practical, this reliance on self-report introduces vulnerability to social desirability bias and limits the extent to which findings reflect what learners actually do with patients. ^13, 14^ Recent work in arts, humanities and empathy education has therefore called for greater use of performance-based assessments that capture learners’ observable behaviours in authentic or simulated clinical scenarios.

Taken together, these developments highlight the need for empathy-focused interventions that (1) use theatre-based experiential methods to rehearse empathic communication in action, (2) are positioned at curricular transition points where empathy may be at risk, and (3) are evaluated with performance-based, behavioural measures rather than self-report alone. Because theatre-based empathy teaching had not previously been incorporated into our curriculum, we developed a structured programme based on existing applied-theatre and communication-skills work and implemented it as a small-scale pilot to explore feasibility and short-term impact before considering wider integration. Breaking bad news is assessed in OSCEs, but there is no dedicated, theatre-based teaching on the empathic aspects of these encounters; this pilot was designed to address that gap immediately before clerkships. We therefore offered a four-week, voluntary theatre-based empathy programme for third-year medical students, delivered alongside the existing curriculum and underpinned by experiential learning principles, using embodied rehearsal, enactment and guided reflection to support empathic communication.

The aim of the present study was to conduct a pilot evaluation of this pre-clerkship programme, examining short-term changes in observable empathic communication behaviours in simulated patient encounters, assessed with a structured observational checklist. We report short-term post-intervention findings intended to inform feasibility, refinement and potential future curricular integration of theatre-based empathy teaching, rather than to provide definitive evidence of long-term effectiveness.

## Methods

### Study design and participants

This was a single-institution, single-group pre–post pilot evaluation of an extracurricular, theatre-based empathy programme for third-year medical students in a six-year MD programme. All 60 third-year students were eligible to participate, but because this was an exploratory pilot designed to assess feasibility, participation was capped at 18 students. To recruit participants, the Dean and programme director convened a meeting for all third-year students and invited them to register for the programme within a two-day period; places were allocated on a first-come, first-served basis. Thirty-nine students registered, and the first 18 were enrolled and assigned to three groups of six.

Attendance at all eight sessions (two per week over four weeks) and completion of both pre- and post-intervention assessments were required for inclusion in the analysis. Six students missed one session each due to personal reasons (mostly short-term illness) and were therefore excluded, leaving a final analytic sample of 12 students. Thus, although a substantial proportion of the eligible cohort expressed interest, the final analysed sample reflected both the planned enrolment cap for this pilot and the strict completion criteria applied for inclusion in the analysis. This study was conceived as an exploratory pilot to assess feasibility and obtain preliminary estimates of change in observable empathic communication. The final sample of 12 students is consistent with published recommendations suggesting that a sample size of approximately 12 participants per group is adequate for pilot work aimed at estimating parameters and informing the design of subsequent larger studies. ^15^ Accordingly, the study was not powered to detect small effects, and all analyses were treated as exploratory.

### Intervention design

The intervention was a four-week programme conducted at Ken Walker International University, Tbilisi, Georgia, comprising two 120-minute sessions per week for each group: one actor-led workshop and one faculty-supervised practice session co-facilitated by the actor. The learning objectives were to support students in recognising and responding to patients’ emotional cues, using verbal and non-verbal behaviours that communicate empathy, and integrating their own emotional responses while maintaining professional boundaries. The programme design drew on Kolb’s experiential learning cycle and applied theatre principles, combining concrete experience in doctor–patient role-play, guided reflection and feedback, conceptual discussion of empathic communication strategies, and repeated enactment of scenarios from both doctor and patient perspectives. ^16^

During the first week, the two sessions served distinct purposes. In the first session, one of the clinical scenarios (advanced cancer with limited prognosis) was introduced and students, working in pairs, conducted 10–15-minute doctor–patient role-plays so that each student enacted both the doctor and patient roles. These untrained encounters, conducted without prior preparation or specific instruction, were used to obtain a baseline measure of “native” empathic communication. In the second session, the professional actor conducted an intensive workshop on basic principles of acting, body language and other non-verbal aspects of communication, to prepare students for subsequent scenario-based work.

From the second week onwards, clinical scenarios were adapted from existing “breaking bad news” OSCE cases used in the main curriculum. The faculty members and the professional theatre actor, who had extensive experience as a simulated patient in OSCEs for more than 10 years, reviewed these cases and selected three (advanced cancer with limited prognosis, a progressive neurological condition in an athlete, and infertility in a young married couple), elaborating them to foreground social, emotional, psychological and economic dimensions. Each weekly scenario formed the basis of both sessions for that week.

Students were assigned to groups of six and worked in rotating pairs. For every scenario, both members of each pair were required to play both roles, doctor and patient, so that all students experienced the encounter from each perspective. Role-plays were conducted sequentially, with one pair performing at a time while the remaining students observed and contributed to feedback. In the actor-led sessions, a brief introduction to the scenario and its communication focus was followed by a short group discussion of the patient’s likely challenges. Pairs then conducted 10–15-minute consultations, each followed by brief feedback, until all students had enacted both roles. The professional actor primarily observed these role-plays and provided detailed feedback on the authenticity and clarity of emotional expression, the credibility of the patient portrayal and the impact of the doctor’s verbal and non-verbal behaviours; when appropriate, he briefly demonstrated alternative ways of handling specific moments. Sessions concluded with a short group debrief.

In the faculty-supervised sessions, the same scenario was revisited. Pairs again rotated through both roles while the faculty member and actor observed. After each encounter, the faculty member provided structured feedback on communication, empathy and professionalism, and the actor added comments on emotional presence and non-verbal communication; brief peer feedback followed. A single faculty member (clinician and communication skills teacher) and the same professional actor facilitated all sessions to maximise consistency, while the wider clinical skills faculty contributed to the initial definition of learning objectives and selection of clinically relevant scenarios. In combination, these components were intended to enable students to rehearse empathic communication repeatedly, experience encounters from both doctor and patient perspectives, and receive multi-source feedback from an experienced actor, peers and faculty.

## Outcome measures

### Performance-based assessment

The primary outcome focused on changes in observable empathic communication behaviours in simulated clinical encounters. This was assessed using the Empathetic Communication Assessment Form developed by Dow et al., ^17^ applied without modification. The instrument groups items into five domains (Empathic Communication, Relating to the Listener, Non-verbal Communication, Verbal Communication and Respect for Dignity), each item rated on a 10-point scale (1 = poor, 10 = excellent). Domain scores were calculated as the mean of items within each domain; an overall score was calculated as the mean of all items.

A baseline assessment was conducted in the first session, before any structured training; each student conducted a 10–15-minute consultation in the doctor role; this first untrained encounter was rated. The post-intervention assessment took place in the final faculty-supervised session, again with each student acting as the doctor in a 10–15-minute consultation.

In line with the programme’s 360-degree feedback design, each performance was rated independently by the faculty member, the actor, at least one observing peer and the student in the patient role. For the primary quantitative analysis, only the faculty and actor ratings were used, as these were most closely aligned with existing assessment practices. Peer ratings were used formatively in feedback but were not analysed statistically.

### Data analysis

Quantitative analyses were conducted using SPSS (version 27.0). For the primary outcome, pre- and post-intervention means and standard deviations were calculated for faculty and actor-rated total and domain scores on the Empathetic Communication Assessment Form. Normality of the pre-post difference scores was assessed using the Shapiro-Wilk test and visual inspection of Q-Q plots. As the distributions were considered sufficiently approximate to normal, paired-samples t-tests were used to examine mean change in scores over time, and Cohen’s d was calculated to estimate within-subject effect sizes. As this was a small pilot study, these analyses were treated as exploratory and intended to provide preliminary estimates of change rather than definitive hypothesis testing. Statistical significance was set at p < 0.05.

### Ethics

The study was approved by the institutional ethics committee of Ken Walker International University (approval number #1-2024/001). All participants received written and verbal information about the study and provided written informed consent. Participation or non-participation had no impact on academic standing. Sessions were conducted under agreed ground rules for confidentiality and psychological safety, and students could pause or withdraw from role-plays at any time. The study adhered to the principles of the Declaration of Helsinki.

## Results

Eighteen third-year medical students enrolled in the programme; six missed one of the eight sessions due to personal reasons (mostly short-term illness) and were excluded to ensure that only students who completed the full programme were included in the analysis. The final analytic sample therefore comprised 12 students (58.3% female; mean age 21.25 years, SD 0.75). None reported prior acting experience. Given this small, self-selected pilot sample, the quantitative findings are interpreted as preliminary estimates of change.

Scores are based on the mean of faculty and actor ratings on the Empathetic Communication Assessment Form. Pre–post increases were observed in all domains of empathic communication ( Table 1). Mean changes ranged from +1.3 to +2.6 points on the 10-point scale, with large within-subject effect sizes (Cohen’s d _x_ ≈ 1.5–2.8).

**Table 1.** Pre- and post-intervention scores on the Empathetic Communication Assessment Form (n = 12).

Domain	Pre-test Mean (SD)	Post-test Mean (SD)	Mean change (Post–Pre)	95% CI for difference	Cohen’s d	p-value
**Empathetic Communication**	6.6 (0.85)	8.8 (0.92)	+2.2	1.6 to 2.8	2.48	<0.001
**Relating to Listener**	6.2 (1.03)	8.8 (1.22)	+2.6	1.9 to 3.3	2.22	<0.001
**Non-verbal Communication**	7.5 (0.98)	9.3 (0.87)	+1.8	1.1 to 2.5	1.56	<0.001
**Verbal Communication**	6.5 (0.96)	8.9 (0.92)	+2.4	1.9 to 3.0	2.80	<0.001
**Respect for Dignity**	7.5 (0.83)	9.0 (0.70)	+1.5	0.9 to 2.1	1.53	<0.001
**Overall**	7.4 (0.88)	8.7 (0.94)	+1.3	0.7 to 1.9	1.47	<0.001

**Figure 1.**
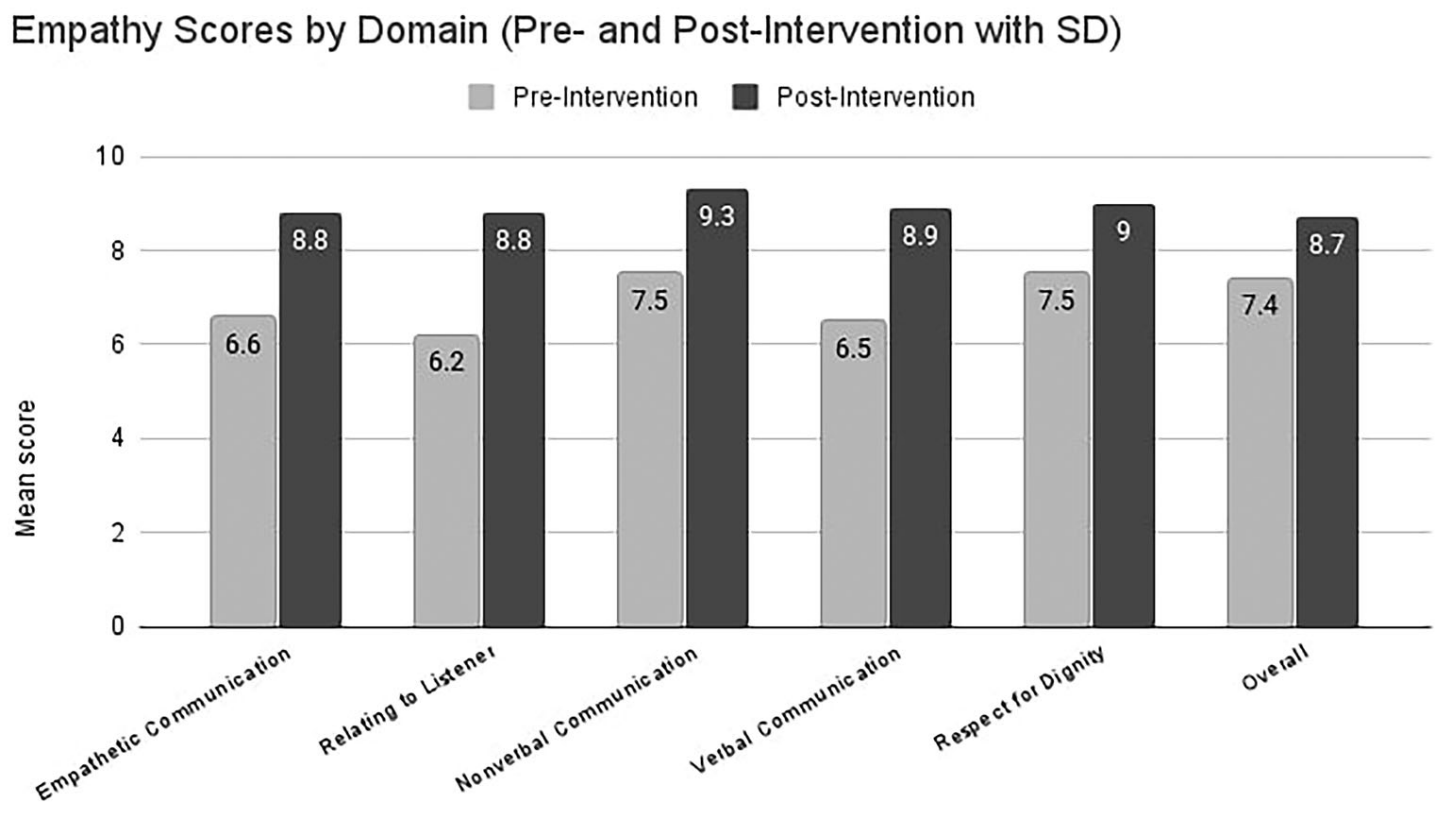
Mean change in empathy-domain scores before and after training. Light-grey bars show the pre-intervention mean (± 1 SD) for each domain in 12 medical students; hatched dark-grey bars show the post-intervention mean (± 1 SD) after the four-week actor-led workshop. Numeric labels above each bar give the exact group mean. Post-intervention scores were significantly higher than pre-intervention in every domain (paired two-tailed t-tests, all p < 0.001).

All 12 participants showed an increase in their overall mean checklist score, with individual improvements ranging from +0.8 to +3.1 points. Exploratory comparisons by gender did not suggest a clear pattern of differential change; given the very small numbers, no firm conclusions can be drawn. Overall, these findings suggest sizable short-term improvements in observed empathic communication behaviours following the pilot programme, but should be regarded as preliminary given the small sample, self-selection and absence of a control group ( Table 1; Figure 1).

## Discussion

In this single-group, pre–post pilot study, a four-week, theatre-based empathy programme for third-year medical students was associated with short-term increases in observed empathic communication behaviours during simulated encounters. Across all domains of the Empathetic Communication Assessment Form, mean scores increased by 1.3–2.6 points on a 10-point scale, with large within-subject effect sizes. On a 10-point rating scale, these changes represent approximately one to two and a half points of improvement per domain. These findings suggest that an extracurricular, actor-led, pre-clerkship intervention may help support the development of empathic communication at a curricular transition point where empathy is known to be vulnerable.

The pattern of change reflects the areas most directly targeted by the programme. Domains showing the largest gains, “Empathetic Communication”, “Relating to the Listener” and “Verbal Communication”, map closely onto elements emphasised in the actor-led workshops and faculty-supervised practice, including attunement to patients’ emotional cues, integration of verbal and non-verbal messages and explicit checking of understanding. The observed improvements in non-verbal communication and in items such as “integration of self” are compatible with the idea that embodied rehearsal, feedback and repeated enactment may help students become more intentional in how they physically and emotionally “show up” in difficult conversations. By requiring students to alternate between doctor and patient roles, the programme also offered structured opportunities for perspective-taking and reflective observation, in line with Kolb’s experiential learning cycle, although the present study did not directly examine mechanisms of change.

These preliminary results align with previous work suggesting that empathy can be fostered through structured, reflective, feedback-rich educational experiences. ^8– 10^ In a forum-theatre study from Angers, medical students showed significant post-session gains on all three Jefferson Scale of Physician Empathy factors, “perspective taking,” “compassionate care,” and “standing in the patient’s shoes,” that is, taking the patient’s position cognitively and affectively. ^9^ Similarly, in our pilot we observed large performance-based improvements in domains such as “Empathetic Communication”, “Relating to the Listener” and, notably, “integration of self,” suggesting that students were not only able to adopt the patient’s perspective in principle, but also to embody that stance in how they presented themselves, responded emotionally and communicated during simulated breaking-bad-news encounters. Within the broader field of arts and humanities in medical education, theatre-based interventions have been reported to enhance communication skills and self-perceived empathy. ^8, 9, 12^ Our findings are compatible with this literature but extend it in two respects. First, theatre-based empathy teaching was deliberately positioned immediately before clerkships, addressing concerns about empathy decline at the transition to clinical training. Second, we evaluated outcomes using a structured observational checklist of communication behaviours, in contrast to much of the existing literature, which has relied primarily on self-report measures. This approach responds to calls in the empathy and arts-in-medical-education literature for greater use of performance-based assessments that capture what learners actually do in clinical encounters. ^10– 14^ The co-designed nature of the programme, developed with a professional actor who also contributed to feedback, resonates with recent recommendations to include the perspectives of artist-practitioners alongside medical educators in curriculum design and evaluation ^6^ and with the AAMC FRAHME initiative’s emphasis on arts and humanities as essential to professional identity formation. ^5^

From a feasibility perspective, the study suggests that a small-group, actor-led programme can be implemented alongside the existing curriculum at the pre-clerkship stage. Interest was high: 39 of 60 eligible students registered within the two-day window for 18 available places, and two-thirds of enrolled students (12/18) completed all eight sessions and assessments. The intervention was delivered using one faculty member and one professional actor across all groups, which may support consistency and, in some contexts, practicality. These observations, together with the observed improvements in checklist scores, have informed ongoing discussions about how a refined version of the programme might be integrated more formally into the third-year communication skills curriculum, accompanied by further evaluation.

Several limitations temper the interpretation of these findings. This was a single-institution pilot with a small, self-selected sample of 12 students; the study was not powered to detect small effects, and the results should be viewed as preliminary estimates of change rather than definitive evidence of effectiveness. Because participation was voluntary and no comparison data were collected for students who did not enroll, we cannot determine whether participants differed systematically from other eligible students in ways that may affect representativeness and generalisability. The lack of a control or comparison group means that alternative explanations, such as maturation, concurrent teaching or Hawthorne effects, cannot be ruled out, and causal inferences about the impact of the programme are not warranted. Assessments were based on simulated encounters with an actor rather than real patients, so the extent to which these behaviours transfer to routine clinical practice is unknown. Ratings were provided by the faculty member and actor who facilitated the programme; raters were not blinded to assessment timing, and inter-rater reliability was not examined, which may introduce bias into the observed score changes. In addition, analyses were restricted to students who attended 100% of sessions. Six enrolled students were excluded because they missed one or two classes due to short-term health issues. Given the brief duration of the intervention, even limited non-attendance may have influenced exposure to key learning components and, consequently, outcome estimates; for this reason, only full completers were included in the final analysis. Finally, the evaluation captured only immediate post-programme outcomes; we did not assess longer-term retention of skills or their impact during clerkships, nor did we collect qualitative data on students’ experiences or perspectives on the mechanisms through which the programme influenced their communication.

Future work should build on these pilot findings using larger, more diverse cohorts and, where feasible, controlled or quasi-experimental designs. This pilot also suggests that the intervention is feasible and acceptable when delivered in a small-group format with a professional actor and faculty facilitation. Nevertheless, implementation at full-cohort level would require greater institutional support, particularly in relation to faculty workload, session scheduling, and access to trained actors or facilitators. Wider rollout is therefore realistic, but would need thoughtful operational planning to ensure that the reflective, feedback-rich character of the intervention is maintained. Longitudinal follow-up into clinical rotations and OSCEs, and ultimately patient-reported outcomes, would help clarify whether observed gains in simulated settings are sustained and clinically meaningful. Mixed-methods approaches incorporating focus groups or interviews with students, faculty and actors could provide richer insight into how participants experience theatre-based empathy training, which components they perceive as most valuable and how such programmes might be adapted for different institutional and cultural contexts. Such work would also contribute to ongoing efforts, exemplified by FRAHME, to build a stronger evidence base for arts- and humanities-informed approaches in medical education.

## Conclusion

This pilot study suggests that a short, theatre-based, pre-clerkship programme co-facilitated by a professional actor and clinician may enhance observable empathic communication behaviours in simulated breaking-bad-news encounters. The findings contribute to emerging efforts, exemplified by the FRAHME framework, to integrate arts and humanities—and in particular theatre and applied performance—into medical curricula in ways that are evaluated using performance-based outcomes. Further research is needed to determine how best to scale, adapt and sustain such interventions to support the development and maintenance of empathy across the continuum of medical training.

## Ethics approval

This study received ethics approval from Biomedical Research Ethics Committee at Ken Walker International University (Approval Number: #1-2024/001). All procedures complied with institutional guidelines and the principles of the Declaration of Helsinki.

## Data Availability

All underlying data, extended materials, and the STROBE checklist are deposited in the Open Science Framework at https://doi.org/10.17605/OSF.IO/U3RBX. All files are available under the Creative Commons Attribution 4.0 International (CC BY 4.0) license. Available through the OSF link above: Empathy domain data: Participant-level pre- and post-scores for five empathy domains (Empathetic Communication, Relating to Listener, Non-verbal, Verbal, Respect to Dignity), with calculated differences and percentage changes. Empathy individual pre-post data: Item-level Empathetic Communication scores for participants (IDs 1–12) before and after the intervention, plus summary statistics. Empathy survey: Image of the 28-item empathy checklist administered to students. Empathy training protocol: Week-by-week outline of the acting-based empathy training programme. STROBE checklist: Completed STROBE reporting checklist. The study follows STROBE recommendations for observational research; the completed checklist is available through OSF.
