# Supplementary material for: Exploring the Impact of Professional Acting on Empathy Development in Medical Students
Source: MedEdPublish (2016). 2026 Apr 6;15:135. Originally published 2025 Sep 19. [Version 3] doi: 10.12688/mep.21228.3 (PMC12996936; doi:10.12688/mep.21228.3)
Supplement: Peer review history

# Reviewer report: Reviewer response for version 3

**DOI:** 10.21956/mep.23097.r45653

**Published:** 2026-04-14

**Authors:** Candace Chow

**Affiliations:** 1 University of Utah, Salt Lake City, Utah, USA

No additional changes requested.

Have any limitations of the research been acknowledged?

Yes

Is the work clearly and accurately presented and does it cite the current literature?

Partly

If applicable, is the statistical analysis and its interpretation appropriate?

Yes

Are all the source data underlying the results available to ensure full reproducibility?

Yes

Is the study design appropriate and is the work technically sound?

Partly

Are the conclusions drawn adequately supported by the results?

Partly

Are sufficient details of methods and analysis provided to allow replication by others?

Partly

Reviewer Expertise:

Qualitative research, mixed-methods research, professional identity

I confirm that I have read this submission and believe that I have an appropriate level of expertise to confirm that it is of an acceptable scientific standard.

# Reviewer report: Reviewer response for version 3

**DOI:** 10.21956/mep.23097.r45655

**Published:** 2026-04-10

**Authors:** Andrea Dillon

**Affiliations:** 1 Adelaide Medical, The University of Adelaide Adelaide Medical School, Adelaide, South Australia, Australia

I am happy with the revisions made to this article. There is one new sentence that should be revised where it refers to the actor as "he briefly". This should be replaced with "the actor briefly".

Have any limitations of the research been acknowledged?

Yes

Is the work clearly and accurately presented and does it cite the current literature?

Partly

If applicable, is the statistical analysis and its interpretation appropriate?

Yes

Are all the source data underlying the results available to ensure full reproducibility?

Yes

Is the study design appropriate and is the work technically sound?

Yes

Are the conclusions drawn adequately supported by the results?

Yes

Are sufficient details of methods and analysis provided to allow replication by others?

Partly

Reviewer Expertise:

Learning and teaching (including assessment) in health professions education

I confirm that I have read this submission and believe that I have an appropriate level of expertise to confirm that it is of an acceptable scientific standard.

# Reviewer report: Reviewer response for version 3

**DOI:** 10.21956/mep.23097.r45654

**Published:** 2026-04-08

**Authors:** Adelina Alcorta-Garza

**Affiliations:** 1 Oncology Service, Autonomus University of Nuevo León, Monterrey, Nuevo León, Mexico

The authors responded by adequately making the requested corrections. I recommend the approval of the article.

Have any limitations of the research been acknowledged?

Yes

Is the work clearly and accurately presented and does it cite the current literature?

Yes

If applicable, is the statistical analysis and its interpretation appropriate?

Partly

Are all the source data underlying the results available to ensure full reproducibility?

Partly

Is the study design appropriate and is the work technically sound?

Yes

Are the conclusions drawn adequately supported by the results?

Partly

Are sufficient details of methods and analysis provided to allow replication by others?

Yes

Reviewer Expertise:

Psychometrics, empathy, personality, and psychodynamics of doctor-patient education, teamwork, and psychosocial factors. Psycho-oncology and palliative care.

I confirm that I have read this submission and believe that I have an appropriate level of expertise to confirm that it is of an acceptable scientific standard.

# Reviewer report: Reviewer response for version 2

**DOI:** 10.21956/mep.22963.r45457

**Published:** 2026-03-17

**Authors:** Adelina Alcorta-Garza

**Affiliations:** 1 Oncology Service, Autonomus University of Nuevo León, Monterrey, Nuevo León, Mexico

Comments to the manuscript titled: Exploring the Impact of Professional Acting on Empathy Development in Medical Students

 A very interesting intervention study, well explained. The background is original and well supported by a solid theoretical foundation. The objective is clearly defined, and the intervention and primary outcome measure are fully described.  There is a limitation regarding sample size: the authors acknowledge that the study was not powered to detect small effects, and all analyses were treated as exploratory.

 I have only the following minor comments on the text.  

 In what city is the institution located?

 Statistical analysis: Please indicate whether the data met the assumption of normality and justify the use of the paired t-test.

 Table 1. I suggest using “mean change” instead of “mean difference”.

 The authors should consider the potential for selection bias, as only 30% of eligible participants agreed to participate. The authors should discuss whether participants differed systematically from those who declined participation, as this may affect the representativeness and generalizability of the findings.

 Discussion and conclusions. They are well written.

Have any limitations of the research been acknowledged?

Yes

Is the work clearly and accurately presented and does it cite the current literature?

Yes

If applicable, is the statistical analysis and its interpretation appropriate?

Partly

Are all the source data underlying the results available to ensure full reproducibility?

Partly

Is the study design appropriate and is the work technically sound?

Yes

Are the conclusions drawn adequately supported by the results?

Partly

Are sufficient details of methods and analysis provided to allow replication by others?

Yes

Reviewer Expertise:

Psychometrics, empathy, personality, and psychodynamics of doctor-patient education, teamwork, and psychosocial factors. Psycho-oncology and palliative care.

I confirm that I have read this submission and believe that I have an appropriate level of expertise to confirm that it is of an acceptable scientific standard, however I have significant reservations, as outlined above.

# Response

**Published:** 2026-03-18

**Authors:** Mariam Rochikashvili

**Affiliations:** Ken Walker International University, Tbilisi, Georgia

We sincerely thank you for your careful review of our manuscript, *Exploring the Impact of Professional Acting on Empathy Development in Medical Students*, and for your constructive comments. We have revised the manuscript accordingly and address each point below.

**Comment 1: Please indicate in what city the institution is located.**

 Thank you. We have added this information in the Methods section and now specify that the study was conducted at Ken Walker International University, Tbilisi, Georgia. **Comment 2: Please indicate whether the data met the assumption of normality and justify the use of the paired t-test.**

 We appreciate this comment and have revised the Statistical Analysis section accordingly. We now state that normality of the pre-post difference scores was assessed using the Shapiro–Wilk test and visual inspection of Q-Q plots, and that paired-samples t-tests were used because the distributions were considered sufficiently approximate to normal. We also clarify that, given the pilot nature of the study and the small sample size, these analyses were treated as exploratory. **Comment 3: In Table 1, use “mean change” instead of “mean difference.”**

 Thank you for this suggestion. We have revised Table 1 accordingly. **Comment 4: The authors should consider the potential for selection bias, as only a subset of eligible participants took part.**

 We agree that this is an important limitation. We have clarified the participant flow in the Methods section by stating that all 60 third-year students were eligible, that participation was capped at 18 because this was an exploratory pilot, that 39 students registered, and that 12 students who completed all sessions and both assessments were included in the final analysis. We have also expanded the limitations section to acknowledge the possibility of self-selection bias and its implications for representativeness and generalisability.

 We hope that these revisions have addressed the reviewers’ concerns and improved the manuscript.

# Reviewer report: Reviewer response for version 2

**DOI:** 10.21956/mep.22963.r45490

**Published:** 2026-03-13

**Authors:** Andrea Dillon

**Affiliations:** 1 Adelaide Medical, The University of Adelaide Adelaide Medical School, Adelaide, South Australia, Australia

The authors have responded to feedback provided by initial reviewers, with the article clearly describing a pilot study utilising professional acting on empathy development of medical students. The article is well written and generally sound, and with amendments would be suitable for indexing.

 While the sample size is small (which is acknowledged by the authors), the results suggest short term improvement in empathy following implementation of the professional acting. The authors clearly describe the limitations of the study. 

 Links to current literature are provided, however some amendments are required. Reference 14: a book is referenced however it suggests [internet] was used - this reference needs to be edited to make it clear that the book was referenced and not just the publisher website, if that is the case. Reference 5 is also lacking sufficient detail and a reference source needs to be added. 

 With regards to the methods, it is unclear whether students observed the student pairs conducting the acting (.i.e.1 pair acting at a time), or whether multiple pairs acted at the same time and the faculty and actor floated between groups. This detail is important and would relate to study feasibility should the pilot be extended to entire cohorts. Inclusion of discussion of whether the authors believe their approach could easily and realistically be extended to an entire cohort  is important. 

 There is mention in the discussion about evaluating "outcomes using a structured observational checklist of communication behaviours rather than relying solely on self-report." As the self-report wasn't included in the analysis, mention of the self-report should be removed as it creates confusion. Correlation of the self-report and assessment of performance would provide an interesting future study.

Have any limitations of the research been acknowledged?

Yes

Is the work clearly and accurately presented and does it cite the current literature?

Partly

If applicable, is the statistical analysis and its interpretation appropriate?

Yes

Are all the source data underlying the results available to ensure full reproducibility?

Yes

Is the study design appropriate and is the work technically sound?

Yes

Are the conclusions drawn adequately supported by the results?

Yes

Are sufficient details of methods and analysis provided to allow replication by others?

Partly

Reviewer Expertise:

Learning and teaching (including assessment) in health professions education

I confirm that I have read this submission and believe that I have an appropriate level of expertise to confirm that it is of an acceptable scientific standard, however I have significant reservations, as outlined above.

# Response

**Published:** 2026-03-18

**Authors:** Mariam Rochikashvili

**Affiliations:** Ken Walker International University, Tbilisi, Georgia

We sincerely thank you for your careful review of our manuscript, *Exploring the Impact of Professional Acting on Empathy Development in Medical Students*, and for your constructive comments. We have revised the manuscript accordingly and address each point below. **Comment 1: Reference 14 appears to cite a book as an internet source, and Reference 5 lacks sufficient detail.**

 Thank you for identifying these issues. We have revised the reference list to ensure that all references are presented with complete and appropriate bibliographic details, including correction of the formatting of Reference 14 and expansion of the information provided for Reference 5. **Comment 2: It was unclear whether students observed one pair at a time or whether multiple pairs worked simultaneously.**

 We appreciate this comment and agree that this detail is important for understanding feasibility. We have clarified in the Methods section that students were assigned to groups of six and worked in rotating pairs, with role-plays conducted sequentially so that one pair performed at a time while the remaining students observed and contributed to feedback. **Comment 3: The manuscript should discuss whether the approach could realistically be extended to the entire cohort.**

 Thank you for this important suggestion. We have added text to the Discussion noting that the intervention was feasible in a small-group pilot format, but that wider implementation would require additional faculty time, scheduling flexibility, and potentially access to more trained actors or facilitators in order to preserve the interactive and feedback-rich nature of the programme. **Comment 4: The mention of self-report in the Discussion created confusion because self-report was not included in the analysis.**

 We agree and have revised the Discussion to remove this ambiguity. The revised text now makes clear that self-report measures are discussed only in relation to prior literature, whereas the present study evaluated outcomes using a structured observational checklist of communication behaviours.

# Reviewer report: Reviewer response for version 2

**DOI:** 10.21956/mep.22963.r45454

**Published:** 2026-03-04

**Authors:** Candace Chow

**Affiliations:** 1 University of Utah, Salt Lake City, Utah, USA

I appreciate the authors' engagements with the suggestions and have no further comments to offer.

Have any limitations of the research been acknowledged?

Yes

Is the work clearly and accurately presented and does it cite the current literature?

Partly

If applicable, is the statistical analysis and its interpretation appropriate?

Yes

Are all the source data underlying the results available to ensure full reproducibility?

Yes

Is the study design appropriate and is the work technically sound?

Partly

Are the conclusions drawn adequately supported by the results?

Partly

Are sufficient details of methods and analysis provided to allow replication by others?

Partly

Reviewer Expertise:

Qualitative research, mixed-methods research, professional identity

I confirm that I have read this submission and believe that I have an appropriate level of expertise to confirm that it is of an acceptable scientific standard.

# Reviewer report: Reviewer response for version 1

**DOI:** 10.21956/mep.22736.r44016

**Published:** 2025-11-05

**Authors:** Candace Chow

**Affiliations:** 1 University of Utah, Salt Lake City, Utah, USA

Overall: I wonder whether this submission better fits the Brief Reports category, given the preliminary results and innovative nature of the intervention. Additionally, while the word count limit for the research submission category is quite high (up to 6000 words), this submission was very short. The authors would have benefitted from using additional words to elaborate on aspects of the study. If this were submitted as a Brief Report, my comments would be different because the guidelines for that submission type are different.

 Introduction: As a reader, I would have liked to understand the “so what?” of the study earlier. While the authors indicate in the first paragraph that empathy is important, I believe the heart of the issue they’re trying to address is sustaining empathy/ensuring it does not decline as medical students move through medical training. Stating this upfront would strengthen the need for the current study. Additionally, while I understand why empathy is juxtaposed with growing use of AI in the first paragraph, I found the sentence about AI distracting from the true issue (teaching medical students how to develop/sustain empathy) and would recommend removing this reference or at least not leading with it. While the authors include many references to studies on empathy, it could be advantageous to also include references to the use of arts and humanities in health sciences education. (AAMC’s FRAHME resources would be a good place to start.)

 Methods: The intervention described is interesting. Could the authors further expand on what the scenarios focused on during each workshop? This would give the reader additional insight on how the students were taught empathy. Were the 60 students invited to participate all the students in the 3 ^rd^ year? This is what I assumed but it could be made clearer. I would have liked to see additional data points. While the comparison of pre- and post- data is promising, it does not provide very much evidence of change given the small sample size. Using a mixed-methods approach and collecting qualitative data through focus groups or interviews (which would have been feasible considering the small N) could have yielded additional data and provided a much more compelling case continuing to use this intervention. In addition, qualitative data would provide insight into *how* this intervention worked.  

 Results: The results are accurately reported, and I appreciate that the authors looked to see whether any gender differences existed. But, as stated above, I would have liked to see more data.

 Discussion: The discussion accurately summarizes the results. It could have been useful to compare the findings to additional literature here.

 Conclusion: Appreciate the authors’ note about further investigation into longitudinal impact.

Have any limitations of the research been acknowledged?

Yes

Is the work clearly and accurately presented and does it cite the current literature?

Partly

If applicable, is the statistical analysis and its interpretation appropriate?

Yes

Are all the source data underlying the results available to ensure full reproducibility?

Yes

Is the study design appropriate and is the work technically sound?

Partly

Are the conclusions drawn adequately supported by the results?

Partly

Are sufficient details of methods and analysis provided to allow replication by others?

Partly

Reviewer Expertise:

Qualitative research, mixed-methods research, professional identity

I confirm that I have read this submission and believe that I have an appropriate level of expertise to state that I do not consider it to be of an acceptable scientific standard, for reasons outlined above.

# Response

**Published:** 2025-12-11

**Authors:** Mariam Rochikashvili

**Affiliations:** Ken Walker International University, Tbilisi, Georgia

Dear Reviewer, 

 We are very grateful for your thoughtful and encouraging comments, especially your suggestions regarding fit, framing and richer description. We have revised the manuscript substantially in response.

1. **Article type and overall length**
**Response: **In line with your recommendation, we have now resubmitted the manuscript as a **Brief Report**. Within this format, we have expanded the text to provide more detail on the rationale, theoretical framing and intervention design, while keeping the paper concise.
2. **Introduction (“so what?” and focus on sustaining empathy)**
**Response: **We have revised the opening paragraphs to foreground more clearly the **risk of empathy decline during clinical training** and the need to *sustain* empathy at key transition points, rather than only emphasising empathy’s general importance. The earlier reference to AI has been removed to keep the focus firmly on empathic development in medical students.
  In addition, following your suggestion, we now explicitly reference work on **arts and humanities in medical education**, including the AAMC FRAHME initiative and a recent scoping review (5,6), to situate the study more clearly within this field.
3. **Methods – scenarios, participants and potential for additional data**
  - **Response: **We have **expanded the Intervention section** to describe in more detail what each set of scenarios focused on (e.g. advanced cancer, progressive neurological disease, infertility), how social–emotional–psychological–economic dimensions were highlighted, and how students alternated between doctor and patient roles within actor-led and faculty-supervised sessions.
  - We have clarified that **all 60 third-year students** were invited and that 18 volunteered on a first-come, first-served basis, with 12 completing all sessions and forming the analytic sample.
  - We fully agree that a **mixed-methods approach with qualitative data** (e.g. focus groups or interviews) would have added important depth about how the intervention worked. While such data were not collected in this first small pilot, we now explicitly acknowledge this as a limitation and highlight mixed-methods evaluation as a key priority for future work.
4. **Results – desire for “more data”**
**Response: **Within the constraints of the Brief Report format and the small pilot sample, we have aimed to make the quantitative findings more informative by:
  - adding **effect sizes (Cohen’s d)** and **95% confidence intervals** to the main results table;
  - clarifying that the findings are treated as **exploratory** and interpreted as preliminary estimates of change.
    We hope this addresses, at least in part, your concern about the strength and interpretability of the quantitative data in a small-N pilot.
5. **Discussion – comparison with additional literature**
**Response: **The Discussion has been expanded to engage more fully with the existing literature. We now:
  - relate our findings to prior studies on empathy training and theatre-based interventions (including forum theatre and MEET);
  - reference work showing gains in “perspective taking,” “compassionate care” and “standing in the patient’s shoes” and connect these to our performance-based improvements (e.g. in “integration of self”);
  - situate our pilot within broader arts-and-humanities/ FRAHME discussions and the call for **performance-based** assessment of empathic behaviours.

  We are grateful for your guidance, which has helped us clarify the focus of the paper, strengthen its theoretical and curricular framing, and align it more appropriately with the Brief Report format.

# Reviewer report: Reviewer response for version 1

**DOI:** 10.21956/mep.22736.r43612

**Published:** 2025-09-30

**Authors:** Eng-Koon Ong

**Affiliations:** 1 National Cancer Centre Singapore Division of Supportive and Palliative Care, Singapore, Singapore; 2 Medical, Assisi Hospice (Ringgold ID: 208643), Singapore, Singapore

this appears to be a simple pre- and post-intervention quantitative education programme evaluation for a acting-based programme for empathy development.  There is a lack of details about the programme, including the exact components and role of the different faculty/actors.  A lack of listed theoretical framework also reduces the rigor of both the programme and the research.  Finally, the small no. of participants renders the quantitative results hard to interpret and may give a false sense of effectiveness.

Have any limitations of the research been acknowledged?

Yes

Is the work clearly and accurately presented and does it cite the current literature?

Partly

If applicable, is the statistical analysis and its interpretation appropriate?

Partly

Are all the source data underlying the results available to ensure full reproducibility?

Yes

Is the study design appropriate and is the work technically sound?

Partly

Are the conclusions drawn adequately supported by the results?

Partly

Are sufficient details of methods and analysis provided to allow replication by others?

No

Reviewer Expertise:

healthcare education, medical humanities, clinical communications, empathy

I confirm that I have read this submission and believe that I have an appropriate level of expertise to state that I do not consider it to be of an acceptable scientific standard, for reasons outlined above.

# Response

**Published:** 2025-12-11

**Authors:** Mariam Rochikashvili

**Affiliations:** Ken Walker International University, Tbilisi, Georgia

Dear Reviewer, 

 We are very grateful for your thoughtful and constructive comments, which have helped us to clarify and strengthen the manuscript. Below we outline the main changes made in response to your points.

1. **Overall framing of the study**
**Response:** We now explicitly describe this work as a *single-group, pre–post pilot study* throughout (title, abstract, Introduction, Methods, Discussion), with the aim of exploring feasibility and estimating preliminary changes rather than demonstrating definitive effectiveness.
2. **Details of the programme and roles of faculty/actors**
**Response:** The **Intervention** section has been expanded to more clearly describe the four-week structure, the specific breaking-bad-news scenarios, students’ rotation between doctor and patient roles, and the respective contributions of the professional actor and faculty member.
3. **Theoretical framework**
**Response:** We now make the theoretical underpinning explicit by linking the programme to **Kolb’s experiential learning cycle** and **applied theatre principles**, and we briefly connect key components of the intervention (role-play, reflection, feedback, repeated enactment) to this framework.
4. **Small sample size and interpretation of findings**
**Response:** We justify the small sample in the context of pilot work (Julious, 2005), describe all analyses as *exploratory*, add effect sizes and confidence intervals to Table 1, and further emphasise in the Discussion that the findings are preliminary and not intended as definitive evidence of effectiveness.

We hope that these revisions address your concerns and that the revised manuscript more clearly reflects the scope and intent of this pilot study.
